# A Comparative Review of Multi-Walled Carbon Nanotube-Reinforced Thermoplastic Petroleum-Based (PET, PBT) and Bio-Based (PLA, PBS) Polyester Systems

**DOI:** 10.3390/ma19143147

**Published:** 2026-07-22

**Authors:** Kashif Ullah Khan, Ferenc Ronkay, Andrea Ádámné Major

**Affiliations:** 1 Doctoral School of Materials Sciences and Technologies, Óbuda University, Bécsi út 96/b, H-1034 Budapest, Hungary; kashif.ullah.khan@nje.hu; 2 Department of Innovative Vehicles and Materials, GAMF Faculty of Engineering and Computer Science, John von Neumann University, Izsáki út 10, H-6000 Kecskemét, Hungary; major.andrea@nje.hu; 3 Institute of Knowledge Technologies, Jászberény Campus, Eszterházy Károly Catholic University, Rákóczi út 53, H-5100 Jászberény, Hungary

**Keywords:** carbon nanomaterials, green nanotechnology, polyesters, MWCNT composites, recycling

## Abstract

**Highlights:**

- Review of MWCNT-reinforced petroleum (PET, PBT) and bio-based (PLA, PBS) thermoplastic polyesters.
- Compares processing methods, mechanical strength, thermal resistance, and electrical conductivity.
- Evaluates the recyclability and circular economy potential of both polyester nanocomposite classes.

**Abstract:**

This review comparatively analyzed MWCNT-reinforced thermoplastic polyester nanocomposites based on petroleum-derived (PET, PBT) and bio-based (PLA, PBS) matrices, focusing on processing methods, mechanical performance, thermal stability, electrical behavior, and recyclability. Optimal MWCNT loadings typically ranged from 0.3 to 3 wt.%; higher loadings induced nanotube agglomeration and deteriorated properties due to poor dispersion and stress concentration. Melt mixing, solution blending, direct compounding, and in situ polymerization were evaluated, and their influence on dispersion quality, interfacial bonding, and scalable manufacturability was discussed. PET exhibited the largest improvements in mechanical and thermal performance (tensile strength and modulus increases >300% in optimized systems); acid or compatibilizer functionalization of MWCNT improved PET thermal stability by approximately 20–50 °C and promoted heterogeneous nucleation. PBT reached optimal reinforcement at 0.3–1 wt.% MWCNT, yielding tensile strength increases up to ~57% alongside increased crystallinity and faster crystallization kinetics. PLA generally showed reduced tensile strength after MWCNT addition unless compatibilized (e.g., via plasticizers or grafting), whereas PBS consistently gained strength, modulus, and crystallinity but experienced reductions in ductility. Electrical percolation thresholds varied widely (0.25–14 wt.%), demonstrating that dispersion quality, nanotube functionalization, and processing route governed conductivity and percolation behavior more than matrix chemistry. Recyclability and circular economy aspects were assessed: while PET/MWCNT systems showed promise for mechanical recycling and property recovery, data on repeated reprocessing, CNT structural integrity, and long-term electrical performance were scarce; PBT recycling studies were limited, and PBS/PLA recycling with retained conductive networks remained underexplored. Based on the comparative analysis, key limitations, critical research gaps, and practical recommendations for processing, compatibilization, and end-of-life evaluation were identified to guide future work aimed at enhancing both performance and sustainability of polyester/MWCNT nanocomposites.

## 1. Introduction

Modern society is based on the use of polymers in everyday products. It is true to state that humans inhabit a polymer-based world. However, storing these materials and employing petroleum-based non-biodegradable polymers in packaging and single-use items are producing problems [1,2,3]. As people become more worried about the chemical industry’s negative effects on human health and the environment, green chemistry (bio-based polymers) becomes more significant in the creation of new processes and products [4].

Carothers, a polymer chemist, discovered polyester in the 1930s. Polyester is known as a condensation polymer since it is formed by a condensation reaction. The reaction consists of joining two monomers to generate a repeating unit and breaking off small molecules, such as water [5]. Furthermore, other compounds, including ammonia, ethanol, and hydrogen sulfide, can also be eliminated [6]. In the early 1940s, World War II was proving to be very destructive, and Germany had to find a suitable replacement for the natural resource-based materials used in commerce and industry due to the Allied blockade. After that, in the 1950s, there was large-scale production of a synthetic polymer material named polyethylene terephthalate (PET) under the Russian name Lavsan [7]. Subsequently, there has been rapid progress in the development of novel production techniques and materials, leading to the emergence of biodegradable polymers as a prominent field of expansion [8].

Petroleum-based polyesters are a family of polymers that are made via the polycondensation of a dicarboxylic acid and a diol. The most common polyesters are PET, polybutylene terephthalate (PBT), polyethylene naphthalate (PEN), and polytrimethylene terephthalate (PTT). These are large-volume polymers used in synthetic fibers, engineering resins, and films. So that petroleum-based polyesters can be handled and used in an industrial environment, the polyesters are melted and compounded with various additives such as plasticizers, UV stabilizers, or flame retardants to make a composite material [9,10,11]. On the other hand, bio-based polyesters are produced from aliphatic diols and aliphatic dicarboxylic acids. The common polyesters are polylactic acid (PLA), polybutylene succinate (PBS), polyhydroxyalkanoates (PHAs), and polyethylene furanoate (PEF). These polyesters are more eco-friendly as they may be degraded quickly by microbes in landfills. Polyesters have been known for a long time and have been employed as insulating films in capacitors and inductors. Recently, their employment has become more ubiquitous, and they are used in capacitors, microelectronic packaging materials, and even explosive detectors [12].

Multi-walled carbon nanotubes (MWCNTs) are a form of carbon and a class of nanomaterial consisting of a cylindrical nanostructure comprising several hollow channels that run along the length of the tube. MWCNTs can be understood by researching their structure, characteristics, and practical applications [13]. In the scientific literature, MWCNTs are generally characterized by supplying the two chiral vector components that explain the structure of any individual nanotube. It is important to highlight that the electrical characteristics of MWCNTs are dependent on how the graphite sheet is rolled up to produce the nanotube. Visual investigation utilizing high-resolution transmission electron microscopes has indicated that flaws can often be observed in the inside wall of an MWCNT. There are various methods for rolling the graphite sheet up to form a MWCNT; otherwise, an interior wall would be impossible to achieve [14]. It also shows that some forms of MWCNT may have superior electrical conductivity than others, as the quantity and type of flaws would influence the overall electronic structure of the nanotubes [15,16].

Nanocomposites are leading the way in material research, as their properties and the area of applications are tremendous. Among several types of nanocomposites, Polyester MWCNT nanocomposites have drawn huge attention from researchers and industrialists because they have great potential in practical applications. Particularly, MWCNTs have a high aspect ratio and extremely good mechanical, thermal and electrical properties. When these materials are incorporated in the polyester matrix, several benefits have been found to date, such as improvements in mechanical properties, thermal stability, and electrical and dielectric properties [17]. One important aspect of these materials that is less researched is the performance and recyclability of such systems once they have reached their useful life. Although there have been many studies carried out on polyester MWCNT nanocomposites, there has been little research done on the recyclability of thermoplastic polyester MWCNT nanocomposite systems [18]. As far as bio-based materials are concerned, one interesting aspect is that these can be further tuned for biodegradability. Studies carried out on PBS/MWCNT nanocomposites in soil have shown that the rate of degradation increases with an increase in the amount of MWCNT-ZnO in the system [19].

In parallel with research related to the performance of petroleum-based systems, rising environmental concerns have increased attention towards bio-based thermoplastic polyesters because of their excellent processability, great thermal stability, and low carbon footprint [20]. Reinforcement of PLA, characterized by excellent tensile strength (50–70 MPa) and modulus (~3 GPa) but poor impact strength and slow crystallization rate [21], is achieved by using liquid-crystalline cellulose fibers with strength values of 1500–1700 MPa [22]. Furthermore, the addition of MWCNT highlighted an increase in flexural strength and stiffness up to 60% and a reduction in the hydrolytic degradation rate in a concentration-dependent manner [23]. Similarly, reinforcement of PBS, a popular matrix for natural fiber green composites [24], can be achieved using the same types of cellulosic filler in the form of pre-treated micro-fibrillated cellulose, increasing the tensile strength and modulus by 123% and 233%, respectively, as well as using coir or cellulose acetate reinforcement providing significant increments in stiffness and strength.

The aim of this review article was to uncover common features and differing behaviors of petroleum-based (PET, PBT) and bio-based (PLA, PBS) polyester nanocomposites reinforced with MWCNT. More specifically, the key objective was to provide an in-depth comparison of petroleum-based polyester MWCNT nanocomposites and their sustainable bio-based analogs with respect to processing methods and their mechanical, thermal, electrical, and recycling properties. Attention was devoted to common reinforcement effects such as heterogeneous nucleation, the formation of conductive networks, and interfacial stress transfer, with special consideration of matrix-specific responses and optimal processing strategies. The review also highlighted current research limitations, including the paucity of direct comparative studies and insufficient investigations on repeated recycling of MWCNT-containing polyester systems. If these aims are achieved, the work is expected to inform future research and support practical applications in areas such as packaging, automotive, and biomedical engineering.

## 2. Types of Polyester Matrices

### 2.1. Petroleum-Based Thermoplastic Polyesters

The term “petroleum-based” means that the material is derived from petroleum or natural gas. Generally, most polyesters are made from petroleum or natural gas. PET, the material from which plastic bottles are made, is the most common polyester. Polybutylene terephthalate (PBT), polyethylene naphthalate, and polyethylene 2,6-naphthalate are three other examples of petroleum-based polyesters [25]. The general chemical formula is HO(CO)R(OCH_2_CHR_2_)_n_OH, and ester groups are often derived from phthalic acid, terephthalic acid, or a combination of the two. The R and R_2_ groups are simply organic moieties such as ethylene, propylene, and butylene. The product of this condensation reaction is named polyester [11,26].

#### 2.1.1. PET

**Figure 1:**
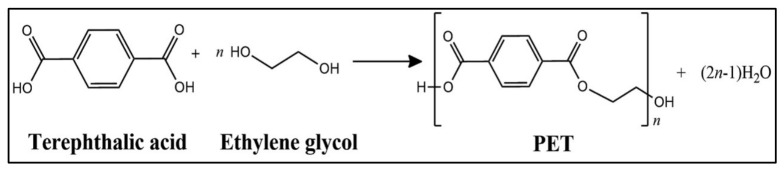
Reaction scheme of PET synthesis.

PET is prepared by a chemical reaction between terephthalic acid and diols (ethylene glycol). The removal of a water molecule from the acid and alcohol produces the ester. The ester then links to another ester unit by a covalent bond created through another dehydration synthesis process. Through these repetitive dehydration synthesis processes, a long and linear chain polymer is produced (Figure 1). This polymer is known as PET [11]. Generally, the glass transition temperature (T_g_) of PET is 70–80 °C, the melting temperature (T_m_) is 250–265 °C, and the density is about 1.34–1.40 g cm^−3^. Industrial PET usually has intrinsic viscosity equal to 0.55–0.85 dL g^−1^ depending on its usage. PET is a polymer with good tensile strength, dimensional stability, transparency, and resistance to dilute acids, oils, and organic solvents. However, it is sensitive to hydrolysis in alkaline media and exposure to high temperatures and water [27,28].

#### 2.1.2. PBT

**Figure 2:**
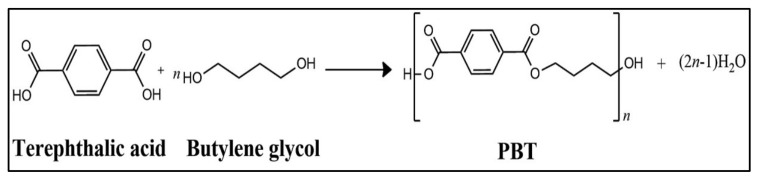
Reaction scheme of PBT synthesis.

PBT is prepared by a chemical reaction between terephthalic acid and diols (butylene glycol) [29]. PBT is structurally very similar to PET, but it has a longer alkyl chain between two terephthalate moieties, which makes the polymer more flexible (Figure 2). However, PBT has a lower melting point (223 °C) than PET (255 °C), so it can be processed at lower temperatures [30,31]. This results in faster crystallization, which is desired for injection molding. PBT typically has a T_g_ of 40–50 °C and a T_m_ of 223–230 °C. PBT has a density of 1.30–1.33 g cm^−3^ and intrinsic viscosity values ranging from 0.8 to 1.2 dL g^−1^ depending on the commercial grade. PBT has high tensile strength, excellent fatigue resistance, low moisture absorption, good electrical insulation properties, and high resistance to hydrocarbons, lubricants, and organic solvents. Nevertheless, prolonged exposure to heat and moisture may cause hydrolytic decomposition of the ester bonds [32]. PBT has already been applied in a plethora of commercial applications like automotive parts, consumer goods that are exposed to high temperature or electrical stress (e.g., egg boilers, hairdryers and oven or iron handles, gear wheels, etc.) [33].

#### 2.1.3. Macromolecular Differences Between PET and PBT

PET and PBT are aromatic polyesters that differ primarily in the length of their glycol segments. PET contains an ethylene glycol unit (–CH_2_–CH_2_–), whereas PBT contains a butylene glycol unit (–(CH_2_)_4_–). The longer aliphatic segment in PBT increases chain flexibility and molecular mobility, resulting in a lower glass transition temperature (T_g_ ≈ 25–50 °C) and faster crystallization rate [34]. In contrast, PET possesses a more rigid macromolecular structure, leading to a higher T_g_ (≈69–80 °C), slower crystallization, and superior dimensional stability [35]. The greater chain flexibility of PBT facilitates chain folding and crystal growth, while PET’s shorter glycol segment restricts molecular motion and enhances stiffness. These structural differences significantly influence processing behavior and end-use properties, with PET favored for high strength and barrier applications and PBT preferred for engineering components requiring rapid molding and good toughness [36].

### 2.2. Bio-Based Thermoplastic Polyesters

Bio-based thermoplastic polyesters are a family of biodegradable and bio-based polymers that can be successfully used for a wide range of applications, including the packaging, biomedical, fiber–textile, and automotive sectors. Being compostable (under industrial composting), many bio-based polyesters can be recycled via reprocessing and re-conversion in final articles, like packaging materials. In general, this family of materials is attracting a lot of attention from the public and the industry and supports the idea of minimizing our plastic footprint and reducing pollution due to plastic materials [37,38,39]. Bio-based polyesters are truly sustainable materials with unique potential for the future. Polyester’s sustainable nature comes from the use of renewable resources and an eco-efficient production process that generates zero waste [40].

#### 2.2.1. PLA

**Figure 3:**
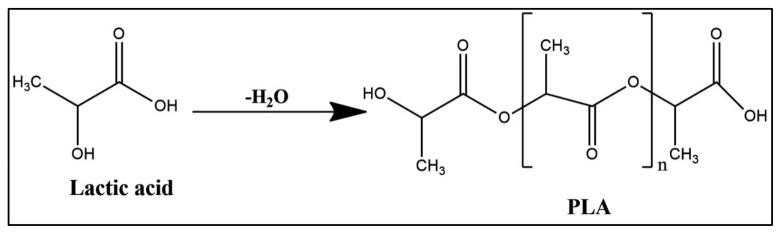
Reaction scheme of PLA synthesis.

PLA is a thermoplastic polyester that is formed through the polycondensation of lactic acid, resulting in a backbone formula of (C_3_H4O_2_)_n_ or [–C(CH_3_)HC(=O)O–]_n_ after water loss (Figure 3). It is essential to conduct this procedure at temperatures lower than 200 °C; if the temperature exceeds this level, the preferred lactide monomer with higher entropy is produced. In industry, high molecular weight PLA is produced via ring-opening polymerization of lactide with the help of catalysts of the tin family, whereas direct polycondensation results in low molecular weight polymer [41]. The term polylactic acid can be misleading, as PLA is not a polyacid but a polyester, which can be confusing [42]. Based on stereochemistry and crystallinity, PLA possesses a glass transition temperature (T_g_) of around 55–65 °C and a melting point (T_m_) of 150–180 °C. The density of PLA is 1.24–1.27 g cm^−3^, and the material provides good rigidity and tensile strength. Nevertheless, PLA demonstrates poor impact resistance, slow crystallization, low heat resistance, and average melt strength, which sometimes necessitates blending or chain extension to ensure processability [43].

PLA, a biodegradable polymer made from renewable resources, shows promise due to the minimal toxicity of its by-products, which eventually break down into CO_2_ and H_2_O [44]. Low energy is needed for its production, leading to a decrease in greenhouse gas emissions. It possesses distinct characteristics that mimic those of PET and polypropylene. Nevertheless, it exhibits an inadequate gas barrier and becomes brittle when fully crystallized.

#### 2.2.2. PBS

**Figure 4:**
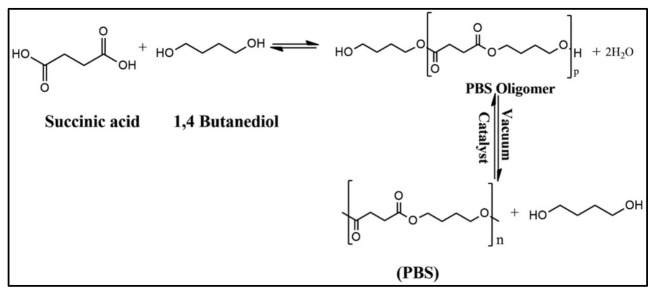
Reaction scheme of PBS synthesis.

PBS is an aliphatic biodegradable polyester and has polypropylene-like properties. The most common method of producing PBS is by directly esterifying succinic acid with 1,4-butanediol. The esterification process consists of two steps (Figure 4). The first step is to esterify excess diol with diacid, resulting in PBS oligomers (with water elimination). The second step is to transesterify these oligomers under vacuum, resulting in a high-molar-mass polymer. This step requires a suitable catalyst (titanium, zirconium, tin or germanium derivatives) [45,46].

PBS has a low T_g_ of around −32 °C and a T_m_ of about 110–120 °C, which allows for great flexibility as compared to PLA. It possesses a density of about 1.24–1.28 g cm^−3^ and moderate melt viscosity that can be further improved via chain extender addition during reactive extrusion. In addition, PBS demonstrates great toughness, elongation at break, impact resistance, and resistance to dilute acids and alcohols while remaining biodegradable via hydrolysis and microbial degradation during composting [43]. Therefore, PBS is widely studied for the development of biodegradable packaging, agricultural mulch films, disposable products, and PLA toughening blends [47].

#### 2.2.3. Macromolecular Differences Between PLA and PBS

PLA and PBS are aliphatic polyesters and biodegradable macromolecules, but their properties differ greatly owing to different chemical structures. Unlike PLA, which comprises the methyl group on its backbone, limiting chain movement, PBS does not have such bulky side groups and is much more flexible, which leads to a low glass transition temperature (T_g_ ≈ 40 °C) [48]. As a result, this macromolecule is characterized by much better ductility, toughness, and crystallization rate compared to PLA, which has a rather high glass transition temperature because of its rigidity (55–65 °C). Another property of PBS is its ability to form well-ordered crystalline phases easily because of its flexible structure, while crystallinity is influenced significantly by stereoregularity in PLA molecules [41]. Therefore, PLA and PBS are distinguished by different mechanical properties, including higher strength and stiffness in PLA and enhanced flexibility and shock resistance in PBS, respectively [43].

### 2.3. Comparative Macromolecular Structure

Unlike PLA and PBS, PET and PBT have significantly different macromolecular structures, which affect all their properties, including thermal, mechanical, and degradability characteristics. Specifically, while PET and PBT are aromatic polyesters, which consist of rigid benzene (terephthalate) rings, PLA and PBS are aliphatic polyesters, which are composed of flexible aliphatic chains (without any aromatic rings) [28,47]. The rigid macromolecular structure of PET and PBT limits molecular chain mobility and makes PET and PBT more thermally stable compared to PLA and PBS. However, both PET and PBT have higher thermal transition points than PLA and PBS [41,43].

In addition, the structural features affect other characteristics of these materials. For example, while PET crystallizes slowly due to the rigidity of its macromolecular structure, PBT crystallizes much faster due to the flexibility of its four-carbon butylene group (in contrast to the two-carbon ethylene group in PET) [32]. PLA crystallizes slowly owing to the presence of asymmetric methyl side groups in its repeating units, and PBS can be easily crystallized owing to the flexibility of its succinate and butylene groups [47]. As a result, PET and PBT are stiffer and have higher tensile strength, dimensional stability, and chemical resistance compared to PLA and PBS.

Moreover, another significant difference between these polyesters lies in their degradability. The rigidity of terephthalate aromatic rings makes PET and PBT very resistant to microbial degradation under normal environmental conditions [27]. Unlike PET and PBT, PLA and PBS have hydrolytically active aliphatic ester bonds that allow chain scission in the presence of moisture, enzymes, and microorganisms, leading to their biodegradation under industrial composting or proper environmental conditions. Thus, PET and PBT are used mostly in engineering applications, while PLA and PBS can be used in sustainable and biodegradable products [49].

## 3. Processing Methods of Polyester MWCNT Nanocomposites

**Figure 5:**
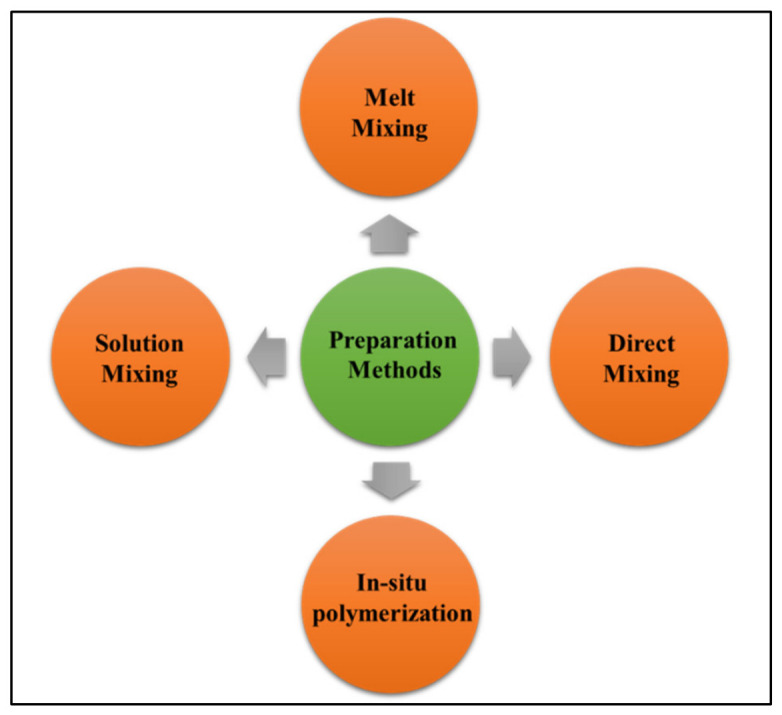
Different methods for preparation of MWCNT polyester nanocomposites.

The major economic and industrial barriers to high-performance polymer nanocomposites are low-cost fabrication and significant scale-up for commercial applications. Currently, four processing approaches are often used to introduce MWCNT into the polyester matrix to create MWCNT/polyester nanocomposites. The common approaches are melt mixing, solution mixing, direct mixing, and in situ polymerization (Figure 5). Melt mixing has been accepted as the simplest and most effective of these processing techniques, particularly from a commercial standpoint, because it allows for the fabrication of high-performance polymer nanocomposites at a low process cost and facilitates commercial scale-up. Furthermore, combining a small amount of pricey MWCNT with standard, cheap thermoplastic polymers offers promising opportunities for improving the physical properties of polymer nanocomposites in a cost-effective manner [50,51]. The choice of fabrication process greatly influences the formation of conducting routes. Therefore, to produce nanocomposites with good electrical, thermal, and mechanical properties, it is necessary to employ several methods, depending on the specific circumstances [52].

### 3.1. Melt Mixing

Among all the methods, melt mixing is the most used method for preparing both types of polyester nanocomposites. Melt mixing is a solvent-free approach for dispersing nanotubes in polyester nanocomposites [53]. The preparation of PET MWCNT nanocomposites was conducted via a melt blending process. Before compounding, the PET polymer and MWCNT were pre-treated in an oven and an acid solution, respectively. The pre-treatment of PET pellets is crucial to increase hydrophilicity and hence the dispersion of MWCNT in the PET matrix. Firstly, the PET pellets were dried in a vacuum oven at 160 °C for 4 h to remove moisture from the pellets. Subsequently, the dried PET pellets were melt-mixed with MWCNT using a laboratory-scale twin-screw extruder in a co-rotating mode. The pre-treated MWCNTs were directly mixed with the dried PET pellets in the extrusion process. The temperature profile of the extruder was set at 240–280 °C from hopper to die zone. The mixing rate of the screw was kept constant at 55 rpm to ensure that the PET and MWCNT were thoroughly mixed to form a homogeneous melt blend in the most efficient manner. Upon completion of the mixing process, the PET MWCNT nanocomposites were extruded as strands and allowed to cool down in ambient air [54,55,56].

**Figure 6:**
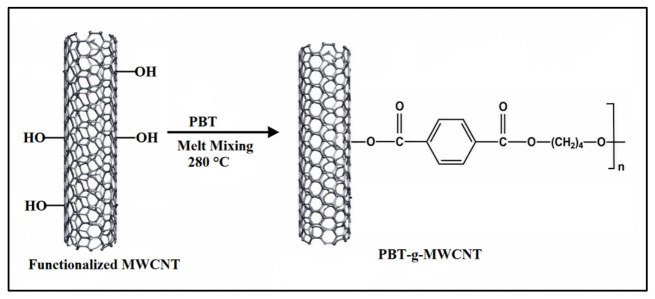
Schematic representation of PBT-MWCNT melt mixing [57].

Furthermore, melt extrusion using a twin extruder produced PBT nanocomposites containing MWCNT-OH (Figure 6). The temperatures of the twin extruder’s feeding zone, melting zone, mixing zone, and exit die were 240, 260, 270, and 280 °C, respectively. Following immediate quenching in a water bath, the melt-mixed composites were chopped with a pelletizer. PBT-g-MWCNT was formed by the in situ transesterification between hydroxyl groups in MWCNT and ester groups in the PBT [57].

**Figure 7:**
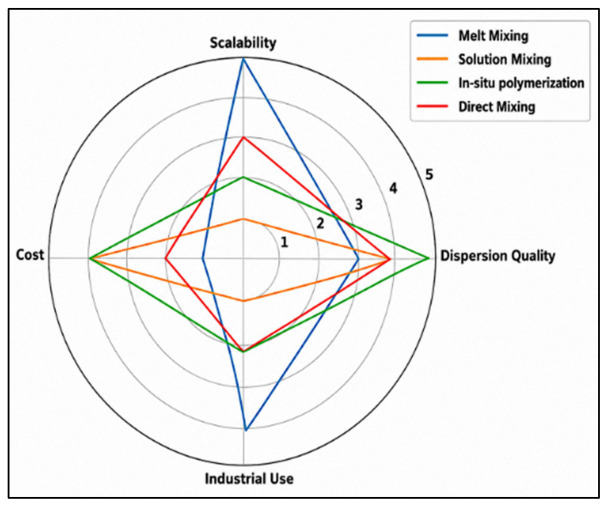
Radar chart comparison of polyester MWCNT nanocomposite processing methods. The numbers 1 to 5 represent a semi-quantitative scoring scale, where higher values indicate greater magnitude or performance for each respective metric.

The synthesis of PBS nanocomposites was accomplished by melt blending in a twin-screw micro-compounder, with a screw rotation speed of 60 rpm, for a duration of 5 min at a temperature of 120 °C. Before the blending process, the PBS was subjected to vacuum drying at 60 °C for 24 h. Following the blending phase, the resulting mixture was transferred to an injection molding machine while in a molten state. The molten compound was then injected into molds at temperatures of 120 °C and 25 °C, respectively [58]. Moreover, the melt mixing method provides several advantages over other methods, but its limitations are nanotube breakage under high shear rates, thermal degradation of bio-based polyesters, and heterogeneous dispersion at high nanofiller content (Figure 7). Higher processing temperatures can be used in the case of PET and PBT but not for PLA and PBS, due to their sensitivity toward thermal molecular weight degradation. While polymer processing with the assistance of solvents may result in the effective dispersion of the MWCNT, it is not recommended due to potential plasticization of the polymer matrix and the difficulties associated with scaling up the process [59].

### 3.2. Solution Mixing

The use of solution mixing as a method of synthesizing nanocomposites has been identified as an effective approach, especially when dealing with biodegradable materials. Recent research indicates that solution blending enables the production of nanocomposite blown films that exhibit improved barrier properties [60]. Similarly, solution mixing has also been used in the synthesis of micro- and nanocomposites of PET-based composites with MWCNTs, through which desired mechanical and electrical properties can be achieved.

Functionalization of MWCNT enables improved compatibility and dispersion within polyester matrices. In this regard, functionalized MWCNTs in PBT-based nanocomposites exhibit higher interfacial interactions leading to improved mechanical properties [61]. Functionalization is mostly carried out using appropriate solution or melt mixing techniques. Additionally, the production of polyester nanocomposites with MWCNTs by using solution mixing provides better dispersion and less agglomeration, but it entails solvent toxicity, which is an environmental concern, as well as high processing cost (Figure 7). In addition to that, solution mixing is primarily used at laboratory scale prior to industrial production [62].

### 3.3. Direct Mixing

The technique of direct mixing is a method involving the direct blending of the nanotubes with the polymer melt without any prior treatment of the surface or dispersion of the solvent. This is one of the techniques used industrially in masterbatch dilution, where a concentrated MWCNT/polymer masterbatch is first produced and subsequently diluted [63]. Integration of MWCNT into polyester matrices through direct mixing processes has received considerable attention among researchers for petroleum-based polyesters because of the improvement in their physical properties. For example, direct melt mixing of PET and carbon nanofibers revealed that processing parameters like screw speed during the process affected the distribution of carbon nanofibers and the properties of the developed nanocomposites [64]. Furthermore, exfoliated graphite nanoplatelets in PET composites showed that direct mixing can blend other types of nanoparticles into polyester matrices [65].

The processing of biodegradable polyesters, including PLA and PBS, through direct melt compounding with MWCNTs has been extensively studied. Mixing PBS and MWCNT produced nanocomposites with excellent barrier and mechanical properties [60]. Similarly, direct mixing of MWCNT with PBS produced nanocomposites that can be useful in applications where structural stability is required [47]. Melt compounding of PLA and MWCNT resulted in blown films with excellent barrier properties, indicating the technique’s applicability in the production of films and packages.

However, direct mixing processes are popular for producing nanocomposites involving MWCNTs and polyesters because of their ease, efficiency, and ability to develop nanocomposites with well-dispersed nanoparticles (Figure 7). Moreover, effective dispersion leads to multifunctional properties owing to the interfacial interactions between the functional groups of MWCNT and the matrix material [60].

### 3.4. In Situ Polymerization

The in situ polymerization technique can be used as an effective tool for adding MWCNT to PET and PLA to produce nanocomposites with improved mechanical properties [66]. Particularly, the creation of PET/MWCNT and PBT/MWCNT nanocomposites is performed through in situ polymerization and provides the required improvements in thermal stability and mechanical strength. Furthermore, in situ polymerization can produce low-toxicity nanocomposites since it allows the in situ synthesis of MWCNTs (Figure 7). The use of this method for creating PET/MWCNT nanocomposites allows increased conductivity to be achieved in a controlled manner [67].

It is important that the in situ technique allows for the production of nanocomposites with improved performance for PLA, particularly strain hardening, foaming, and higher crystallization rates. Due to these properties, PLA-based nanocomposites can be used in more applications than pure polymer due to improved mechanical characteristics [68]. Moreover, PBS-based nanocomposites with high-aspect-ratio MWCNT can be produced using in situ polymerization due to the high efficiency of this approach. Since it is vital to ensure high dispersion and improved interfacial adhesion of MWCNT in PBS nanocomposites, in situ methods are often preferred for this task [69]. Overall, the in situ polymerization technique seems to be critical in the manufacture of MWCNT-reinforced polyester nanocomposites due to its benefits regarding dispersion, bonding, and mechanical performance [70]. The use of such techniques is limited due to complex processing, increased processing cost, and limited industrial use (Figure 7).

## 4. Mechanical Properties

Testing the mechanical properties of the nanocomposites can reveal the impact of incorporating MWCNT fillers into pristine PET [71]. The addition of a small quantity of MWCNT enhanced the mechanical properties of PET/MWCNT nanocomposites due to the reinforcement provided by the high aspect ratio of MWCNTs and their even distribution within the PET matrix [72].

Kim et al. discovered that the inclusion of 1 wt.% of MWCNT resulted in a 10–15% increase in tensile modulus and around a 10% increase in tensile strength [73]. In contrast, G. Santoro et al. achieved similar outcomes with a concentration of only 0.1 wt.% [53]. The addition of 0.1 wt.% MWCNT to the PET matrix increased the rigidity of the polymer. However, it also decreased the material’s ability to stretch before breaking and consequently lowered its toughness, particularly in the composites prepared by direct extrusion. The tensile strength of the composites fabricated with melt compounding followed by extrusion exhibited an increase in comparison to pure PET [59]. Furthermore, H. U. Zaman et al. found that the addition of 0.5 wt.% functionalized c-MWCNT to PET nanocomposites significantly enhances their mechanical properties. Specifically, compared to neat PET, the tensile modulus is enhanced by approximately 15%, and the stress at break is improved by approximately 63%. Given the enhanced mechanical capabilities and lower cost of the generated PET/c-MWCNT nanocomposites compared to polymer nanocomposites with a high c-MWCNT content, it is reasonable to anticipate that these materials will have significant applications in future industries [71,74].

Studies by S. A. Awad et al. [75] employed direct mixing to create PET/MWCNT composites. The MWCNT material was dispersed into PET using an ultrasonic mixing process. The results of elongation at break show that a larger value of MWCNT content restricts the rise in elongation at break to 8.6%, as opposed to pure PET’s 15.5%. Thus, 1 wt.% MWCNT can increase mechanical properties when compared to other MWCNT contents and pure PET [75]. Additionally, Jun Y. Kim [76] stated that adding carbon nanotubes (CNTs) to PBT nanocomposites greatly enhanced their mechanical properties, especially when using lower CNT concentrations. This improvement is attributed to CNTs’ high aspect ratio and extensive surface area, which facilitate the effective transfer of loads from the polymer matrix to the nanotubes. The addition of CNTs into the PBT matrix indicated an improvement in the flexural modulus of the PBT nanocomposites and an enhanced ability of the PBT nanocomposites to maintain the high stiffness caused by CNTs.

In the study by E.Y. Choi et al., PBT composites with PBT-grafted MWCNT were produced through the melt extrusion process. During melt extrusion, the PBT-grafted MWCNTs were created through in situ transesterification between the –OH groups in the MWCNTs and ester groups in the PBT. The tensile strength and modulus of the PBT composite increased with the amount of MWCNT content in the composite. Additionally, the PBT/PBT-g-MWCNT composite exhibited greater tensile strength and modulus compared to the PBT/pristine MWCNT composite at the same MWCNT content [57]. Additionally, F. Ania et al. investigated the micromechanical properties of PBT composites with a small amount of MWCNT nanotube content and temperature variations. Even though there were few filler options, the MWCNT composites show an increase in hardness from 143 MPa for pure PBT to around 160 MPa with just 0.2% CNTs. A leveling-off hardness value was achieved with a higher concentration of MWCNT (0.35%). There is no significant difference in the microhardness data between materials with NH_2_ groups on the surface and untreated MWCNT. In this instance, the surface alteration does not appear to change the micromechanical characteristics [77].

R. Deshpande et al. studied how the better dispersion of CNTs within the PBT matrix contributed to an increase in the composite’s hardness. The decreased MFI value of the functionalized CNT-reinforced composite suggested the creation of a network of interconnected pristine CNTs. Nevertheless, the inclusion of functionalized CNTs caused the composite to become brittle, leading to a decrease in tensile strength and elongation of the composites. While wearing, the occurrence of peeling and scuffing in pure PBT was reduced by adding CNTs but increased with functionalized CNTs due to the higher hardness of PBT after incorporating functionalized CNTs [78].

O. Saligheh et al. demonstrated that adding 1 wt.% of MWCNT to PBT nanofibers significantly increased the specific strength from 163.5 Ncm/g to 220 Ncm/g, and the specific modulus from 304.1 Ncm/g to 358.9 Ncm/g compared to neat PBT nanofibers. The strength and stiffness of PBT/MWCNT composite nanofibers increased in comparison to neat PBT nanofibers. The addition of a small amount of MWCNT (0.5 wt.%) to PBT composite nanofibers increased their elongation at break, but when the amount was increased to 1 wt.%, there was a gradual decrease in elongation at break compared to the 0.5 wt.% nanofibers. The enhancement of the tensile strength and specific modulus of composite nanofibers suggests that the composite nanofibers are more durable and less likely to deform [79]. The reason might be the increased crystallinity and strong alignment of molecular chains in composite nanofibers containing 1 wt.% nanotubes. With higher MWCNT loading, the specific strength and modulus decreased; they tended to clump together, leading to reduced alignment with the fiber axis and weaker bonding with polymer chains. This prevented the transfer of load to the nanotubes, ultimately lowering the tensile strength [80,81].

S. Chopra et al. [82] found that the mechanical strength of PBT/CNT nanocomposites slightly improved up to 0.3 wt.% of CNT. Furthermore, the properties decreased to the point of being equivalent to those of a pure polymer or possibly even lower. There was a significant 122% increase in the elongation at break of PBT 0.3 wt.% compared to pure PBT. It is widely recognized that adding small amounts of CNT to PBT can enhance the mechanical characteristics of the nanocomposites. This is due to the nano-reinforcing impact of CNTs, which is a result of their significantly greater aspect ratio and even distribution within the polymer matrix. When the applied load surpasses the polymer matrix’s elastic deformation stress, the CNTs create a stress transfer effect because of their interconnected network. This improves the toughness of the polymer matrix by stopping matrix degradation [83].

**Figure 8:**
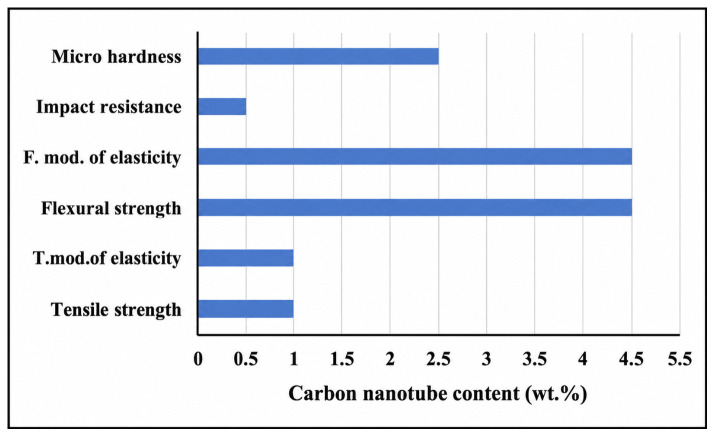
The mechanical properties of PLA/MWCNT nanocomposites with different contents (wt.%) [84].

N. Vidakis et al. created PLA/MWCNT nanocomposites using filler loadings of 0.5, 1.0, 2.5, and 5.0 wt.% through a process of melt mixing and filament extrusion. They thought the most effective reinforcement mechanism would ideally come from a 5.0 wt.% filler loading for each of the various mechanical attributes. Nonetheless, this is only true for the flexibility characteristics and notably not for the tensile, impact, and microhardness traits, as a decline effect has been noted for filler loadings exceeding 1.0 wt.% (Figure 8). This may be more specifically traced back to the presence of CNT aggregates at 5.0 wt.%, which have a detrimental impact on the tensile, impact, and microhardness properties of the PLA matrix [84].

J.H. Lee et al. analyzed the surface mechanical characteristics of PLA/MWCNT nanocomposites through nanoindentation. They found that the stiffness of MWCNTs improved the surface modulus and hardness, thereby enhancing crystallinity via the α′-to-α-phase crystal transition of PLA [85]. Furthermore, Lucia Fama et al. developed a film using PLA reinforced with MWCNTs using a remarkable method to achieve the best filler distribution in the material. MWCNT underwent functionalization through a Fenton reaction to generate hydroxyl (OH) and carboxyl (COOH) functional groups on their surfaces. These modifications allowed for exceptional scattering, a lack of gaps, and very strong bonding between the filler and the PLA. A significant discovery was made when the maximum tensile strength increased by 20% with just 0.10 wt.% of functionalized MWCNT [86].

MWCNT-reinforced PLA/LNR (liquid natural rubber) composites were fabricated by Adilah M. Ali et al. using the melt blending method. Mechanical tests indicate a notable enhancement in tensile strength and tensile modulus at 3.5 wt.% of MWCNT in comparison to pure PLA/LNR. The elongation of PLA nanocomposites decreases with an increase in MWCNT content. In addition, when the ultrasonic energy method was used, a uniform dispersion of MWCNT within the PLA/LNR matrix was attained along with robust bonding between MWCNTs and the matrix [87]. Furthermore, Amirian et al. [88] studied how MWCNT-g-PLLA impacted the mechanical characteristics of PLLA. Mechanical tests indicated that the PLLA/MWCNT-g-PLLAs composites had higher ultimate tensile strengths and elongations at break compared to neat PLLA, with values of 55.8 MPa and 285%, respectively, up from 37.9 MPa and 157% [88].

Furthermore, a study by Sinha Ray et al. demonstrated that the mechanical properties of PBS improved significantly with the incorporation of MWCNTs. The storage flexural modulus increased 88% at room temperature for pure PBS (0.64 GPa) compared to the nanocomposite (1.2 GPa), and the tensile modulus as well as thermal stability of PBS improved moderately after the formation of nanocomposites with 3 wt.% MWCNT [89]. Moreover, L. Tan et al. successfully prepared a blend between PBS and functionalized CNTs. The stress–strain curves for PBS and PBS nanocomposites show that the addition of CNTs significantly increases the elastic modulus of PBS due to the inclusion of rigid components in the relatively soft matrix. The tensile strength for PBS slightly increases with increasing CNT content. When compared to the 1% blend nanocomposite, the 1% hydrolyzed nanocomposite shows higher tensile strength due to improved dispersion of CNTs in the PBS matrix following hydrolysis [90].

Shih et al. found that E’ (storage capacity) for PBS/CNT nanocomposites increased with increasing (pure u-CNT) content as the temperature increased. This suggests that CNT-C18 increases the stiffness of the nanocomposite. However, E’ initially increased with increasing u-CNT content but decreased with increasing u-CNT by 3 wt.%. In general, when u-CNT is higher than 1.5 Wt.%, aggregation occurs in the polymer matrix and poor mechanical properties are observed. Furthermore, the improvement of mechanical properties of the CNT-C18 system at various temperatures is more important than that of u-CNT systems. The increase in E’ for nanocomposites with 3 wt.% u-CNT was 84% at 25 °C, while the increase in E’ at nanocomposite 25 °C was 120% with 3 wt.%. This suggests better dispersion of CNT-C18 in the PBS matrices compared to u-CNT [91].

### Comparative Mechanical Properties

The PET and PBT polyesters have more inherent mechanical stability than the other two because of their aromatic structures, whereas the aliphatic polyesters, i.e., the PLA and PBS, are more prone to trade-offs between stiffness and ductility after MWCNT addition. PET is the most dependent polyester matrix on the processing procedure among the four (PET, PBT, PLA, PBS). The conventional melt compounding procedure leads to increases in the tensile modulus but decreases in tensile strength and elongation because of stress concentrations around the nanotube agglomerates [92]. Melt spinning followed by fiber drawing leads to a simultaneous increase in tensile modulus and strength exceeding 300% [93]. The mild oxidation of the MWCNT surfaces leads to an enhancement in tensile strength and ductility through the improvement in interfacial stress transfer, whereas too much oxidation damages the nanotubes, causing brittleness of the composite [94].

In contrast to PET, PBT has a particular optimal reinforcement threshold at very low concentrations of MWCNT (0.3–1 wt.%). The MWCNT content up to 0.3 wt.% results in an increase in tensile strength of about 57%. This happens because MWCNTs are efficient crystallization nucleating agents in PBT, increasing matrix crystallinity [95]. Similar improvements in tensile, flexural, impact, and tribological properties were achieved by a MWCNT content of about 1 wt.%. After this value, further addition of nanotubes does not cause improvements because of agglomeration [96]. In an impact-modified PBT blend, MWCNT restores stiffness while simultaneously doubling the impact strength compared to the neat matrix, overcoming the trade-off between stiffness and toughness introduced by elastomeric modifiers [97]. However, the current studies concerning PBT are mostly limited to blends; therefore, the reinforcement mechanisms in the neat matrix are unknown (Table 1).

As compared to PET and PBT, PLA has the least favorable inherent response to MWCNT addition. The composites based on unplasticized PLA are characterized by a reduction in tensile strength and ductility despite high increments of flexural modulus due to poor stress transfer by nanotubes [98]. The solvent-cast PLA films exhibit a gradual decrease in tensile strength in the range of 0.5–5 wt.% MWCNT, although elongation at break and crystallinity increase [99]. On the contrary, the PET matrix allows for an increase in tensile strength and ductility through the surface functionalization of nanotubes. Meanwhile, PLA needs plasticization as the compatibilization technique. The use of malic acid oil helped to compensate for the losses in mechanical properties caused by MWCNT addition and increased tensile strength by about 20% compared to neat PLA. Polyethylene glycol (PEG) as a plasticizer increased both tensile and flexural properties even at a low content of 0.15 wt.% MWCNT (Table 1) [100,101].

**Figure 9:**
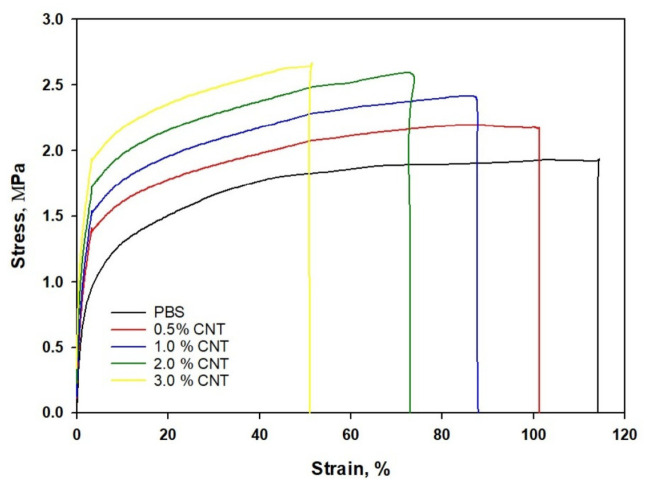
The mechanical curve of MWCNT/PBS electrospun material [102].

Among the four matrices, PBS demonstrates the most regular reinforcement behavior. The electrospun PBS fibers filled with 3 wt.% MWCNT had higher tensile strength and modulus (Figure 9), crystallinity, and thermal stability, although elongation was reduced by 25% (Table 1) [102]. Similar trends were found in melt-compounded bio-PBS composites where tensile strength and stiffness increased along with tribological properties and ductility dropped by 1.5–2-fold [103]. In PLA/PBS blend membranes, the optimal content of 0.5 wt.% MWCNT raised tensile strength by about 30% along with the increase in crystallinity and modulus (Table 1) [104].

There are still several gaps in the research that require filling. The majority of existing PBT studies concentrate on polymer blends, thus making a direct comparison with PET, PLA, and PBS impossible. At the same time, no comparative investigations of the four polyesters using identical types of MWCNTs, identical dispersion procedures, and identical process parameters have been conducted. In addition, the orientation-dependent reinforcement approaches that have been found to be very effective for PET are relatively neglected in PLA and PBT, while long-term stability, fatigue behavior, and structure-property relationships under service conditions are poorly investigated. This would help to develop general rules for designing high-performance composite materials based on MWCNT-reinforced polyester.

**Table 1:** Mechanical properties of different polyester MWCNT nanocomposites.

Matrix	Filler Type	wt.%	Method	Tensile Strength	Tensile Modulus	Flexural Strength	Flexural Modulus	Impact Strength	Main Findings	Ref.
PET	MWCNT	0–2	Melt compounding	46 → 25 (↓ ~46%)	1073 → 1303 MPa (+21.5% at 2 wt.%)	Not reported	Not reported	Not reported	Decline can be attributed to CNT agglomeration limiting effective load transfer at higher loadings.	[92]
PET	MWCNT	0–5	Melt spinning	↑ ≥300% (tensile stress)	↑ ≥300% (elastic modulus)	Not reported	Not reported	Not reported	Post-drawn PET/MWCNT melt-spun fibers showed >300% gains in both stiffness and strength	[93]
PET	MWCNT -COOH	0.5	Melt mixing	↑ 55%	Not significant	Not reported	Not reported	Not reported	Mild oxidation of MWCNT before melt mixing improved PET tensile strength and ductility	[94]
PBT/PC Blend	MWCNT	0.15–0.45	Melt mixing	54 → 85 (+57% at 0.3 wt.%)	+~60% (vs. blend)	Not reported	+~80% (vs. blend)	Improved at 20% PC content	MWCNT acted as a strong nucleating agent in the PBT matrix, raising crystallinity and stiffness	[95]
PC/PBT	MWCNT	1	Melt compounding	+66%	+52%	+533%	+41%	+119%	At 1 wt.% MWCNT, the PC-PBT blend showed simultaneous improvement in mechanical properties; higher loadings gave diminishing returns.	[96]
PLA	MWCNT	0.5–1	Melt mixing	~50.9 → 52.6 (elongation ↓ 2.30% → 1.57%)	stiffness ↑	84.32 at 0.75 wt.% (+60% vs. neat)	3.00 → 3.36 (+12%)	Not reported	MWCNTs preferentially enhanced flexural strength/stiffness; 1 wt.% showed a slight drop from agglomeration	[98]
PLA	MWCNT + (MLO plasticizer)	0.5, 1 (+5 phr MLO)	Melt compounding	50.7 (neat) → −50% w/o plasticizer; +~20% with MLO	1366.8 MPa (neat)	93.83 (neat) → −60% w/o plasticizer	2657.51 MPa (neat)	19.75 kJ/m^2^ (neat, Charpy)	Unplasticized MWCNT composites lost 50–60% of neat-PLA strength; adding 5 phr MLO plasticizer reversed this, giving ~20% higher tensile strength than neat PLA.	[100]
PLA/PEGplasticized	MWCNT	0.15 (+6 wt.% PEG)	Two-roll milling	↑ to 43.8 (vs. plasticized PLA)	Not reported	↑ to 81.4 (vs. plasticized PLA)	Not reported	Not reported	A very small MWCNT loading (0.15 wt.%) in PEG-plasticized PLA raised both tensile and flexural strength	[101]
PBS	MWCNT	0.5–3	Electrospinning	+2.61	+0.72 GPa	Not reported	Not reported	Not reported	MWCNT raised tensile strength/modulus, while elongation at break fell ~24.5%.	[102]
PBS/PLA Blend	MWCNT (masterbatch dilution)	0.5	Solution casting	9.3 → ~12.1 MPa (+30%)	+13%	Not reported	Not reported	Not reported	MWCNT addition to a PLA/PBS gas-separation membrane increased tensile strength and elastic modulus, while reducing elongation at break by ~10%	[104]

“→” indicates the effect of the MWCNT modification on the value of the given property. “↑” indicates an increase in the given property. “↓” indicates an decreasein the given property.

## 5. Thermal Properties

Kashiwagi et al. discovered that CNT layers provided an effective barrier during thermal degradation, insulating polymers and reducing weight loss rates of degradation products, leading to enhanced thermal stability of polymer nanocomposites [105]. A small quantity of MWCNT proved beneficial by acting as efficient thermal decomposition-resistant nano reinforcements in the PET matrix, thereby improving the thermal stability of the PET/MWCNT nanocomposites. The enhanced thermal stability in PET/MWCNT nanocomposites is believed to be due to a physical barrier effect from the MWCNT, stopping volatile products from escaping and slowing down thermal decomposition in the polymer nanocomposites [73,106].

According to Sameer A. Awad et al., [75] the inclusion of MWCNT in pure PET somewhat increases thermal stability because interfaces between multi-functionalized MWCNTs and pure PET reduce brittle behavior due to the high crosslinking of pure PET. The higher the proportion of MWCNT fillers in pure PET, the greater the thermal stability because of the high crosslink density between pure PET and MWCNT. For concentrations below 1 wt.% nanotubes, the influence of the nanotubes on thermal stability is minimal, with just a modest increase observed in the composite with the highest amount of MWCNTs produced [75]. Furthermore, Yoo et al. [107] used a melt extrusion procedure to create PET/MWCNT composites with a nanotube concentration of 3 wt.%. They found that both pure and chemically modified nanotubes resulted in a T_c_ rise of around 158 °C. Another study by Tzavalas et al. [108] found that addition of 0.1 wt.% MWCNT through melt mixing increased the T_c_ of PET by around 128 °C. However, in this case, the temperature increase was more than double. This is due to the CNTs being dispersed more efficiently in the matrix, resulting in a larger surface area for crystal nucleation. Moreover, H. U. Zaman et al. [71] found that incorporating c-MWCNT into the PET matrix has a significant effect on the non-isothermal crystallization behavior of PET nanocomposites, as the C-MWCNT dispersed in the PET matrix can effectively act as strong nucleating agents, resulting in enhanced crystallization of the PET nanocomposites via heterogeneous nucleation.

Y. Gao and coworkers effectively carried out chemical modification of the original MWCNT using maleic anhydride. The enhanced interaction between f-MWCNT and PET chains leads to the successful dispersion of f-MWCNT in the PET matrix. The cold-crystallization and melt-crystallization behaviors of quenched samples both indicate that f-MWCNT promotes nucleation during PET crystallization. The peak temperature of cold crystallization decreases, while the peak temperature of melt-crystallization increases as the f-MWCNT content in nanocomposites increases. The researchers compared the non-isothermal crystallization kinetics of pure PET with PET/f-MWCNT nanocomposites, demonstrating the acceleration of PET crystallization by f-MWCNT in the nanocomposites [109].

D. Bikiaris et al. discovered that incorporating MWCNT during PET polymerization altered most of its characteristics. As the quantity of MWCNT increased, they functioned as versatile agents in the preparation of lower-molecular-weight PET macromolecules. The addition of MWCNT to the PET matrix enhanced thermal decomposition by increasing the decomposition temperature for nanocomposites with up to 1 wt.% MWCNT. The activation energies for different values of a were calculated using the Ozawa, Flynn, Wall, and Friedman iso-conversional methods, showing that all nanocomposites, except the 2 wt.% MWCNT one, had higher activation energies than pure PET [110]. DSC shows that the MWCNTs function as nucleating agents and highlights matrix plasticization during processing [59].

Jun Young Kim observed that adding CNT to the PBT matrix does not significantly impact the T_g_ and T_m_ values of the PBT nanocomposites. The T_c_ values showed a notable rise when CNT was added, which was especially noticeable at lower CNT concentrations. This outcome shows that CNTs are effective nucleating agents for PBT crystallization, improving the crystallization of PBT nanocomposites when CNTs are present. Additionally, the rise in the crystallization temperature of the PBT nanocomposites as the CNT content increases, alongside the reduction in the supercooling degree (DT) for crystallization with higher CNT content, indicates that CNTs may serve as potent nucleating agents in the PBT matrix, ultimately improving PBT crystallization. Hence, adding a small amount of CNT to the PBT matrix can improve the crystallization of the PBT nanocomposites by promoting heterogeneous nucleation. The same findings have been noted in CNT-filled polymer nanocomposites, specifically the faster formation of crystals due to the addition of CNTs causing heterogeneous nucleation [76,111]. Furthermore, E.Y. Choi et al. [57] found that the thermal degradation characteristics of MWCNT-OH were like those of pristine MWCNT. The level of PBT attached to MWCNT-OH could vary depending on the amount of MWCNT-OH present in the composites.

Moreover, Revati Deshpande et al. [78] stated that the alterations in the melting temperature (T_m_) values of the specimens are not highly notable. Conversely, the PBT-CNT composites showed a rise in crystallization temperature (T_c_) of nearly 7 °C. Furthermore, the percentage of crystallinity in PBT rose when CNTs, whether pristine or functionalized, were added. This shows that CNTs serve as nucleating agents in the PBT matrix, thereby enhancing the polymer’s nucleating ability, as seen in higher crystallization temperatures. The elongated fibrous shape of CNTs creates perfect locations for polymer chains to anchor, leading to simpler crystal growth with the presence of thermal energy [112].

Composite nanofibers of PBT/MWCNT were effectively produced by O. Saligheh [81] using the electrospinning method, resulting in a random fiber web. The composite nanofibers made of PBT/MWCNT exhibited greater crystallinity compared to the nanofibers made of pure PBT, and the level of crystallinity rose as the amount of MWCNT in the composite increased. The PBT/MWCNT composite nanofibers with 1 wt.% nanotubes have a crystallinity of 39.76%, which is approximately 11% higher than that of the neat PBT. The presence of CNTs may result in higher crystallinity, suggesting that nucleation of PBT crystallization is taking place in the electrospun composite nanofibers [80]. J.H. Lee et al. [85] examined the thermal properties of PLA/MWCNT nanocomposites with DSC. They observed that the melting peaks of PLA decreased to a lower temperature when MWCNTs were present, indicating a potential interaction between the two materials [113].

**Figure 10:**
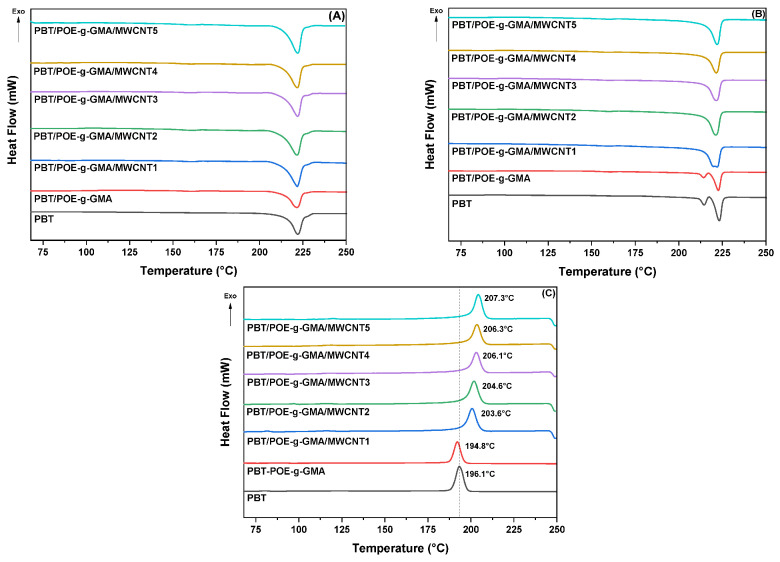
DSC curves for the first heating (**A**), second heating (**B**) and cooling (**C**) of the studied materials [97].

Direct thermal characterization of MWCNT-filled PBT is far less abundant in the literature compared to that on PET composites, and the most comprehensive data set currently available [97] was obtained not for neat PBT but for an impact-modified PBT/POE-g-GMA composite blend, which limits the comparability of this system with neat PBT. In any case, the data on DSC analysis are useful for the distinctive double melting endotherm of neat PBT. The formation of two different lamellar structures during cooling was absent in the presence of MWCNTs, similarly to the nucleation-induced uniformity of the PET/MWCNT composite system. The temperature of crystallization increased by up to 11.2 °C, while crystallinity increased from 24.5% to almost 39%, thus indicating again the nucleating rather than merely reinforcing effect of nanotubes. The heat deflection temperature of the polymer increased from 51 °C to approximately 55 °C, which is likely of greater industrial importance than crystallization from DSC (Figure 10).

The PLA/MWCNT nanocomposites showed a decreased cold crystallization temperature, indicating the nucleating effect of MWCNT. Unexpectedly, there were weak exothermic peaks seen before the endothermic melting peak of the PLA/MWCNT nanocomposites, which were enhanced by the presence of MWCNT [114,115,116]. Following heating to 120 °C and the cooling process, two distinct peaks were observed during melting. This phenomenon can be attributed to the α′-to-α phase transition and the reorganization of the α-phase during recrystallization. The presence of various crystal formations can lead to several melting points [116]. In theory, the transition from α′ to α is typically a release of heat, whereas the process of melt recrystallization of the α-phase crystal created during annealing requires the input of heat [117]. The PLA/MWCNT nanocomposites exhibit a more noticeable second melting peak, whereas the neat PLA shows a more prominent first melting peak. This finding also backs the concept that MWCNT triggers a transition from α′ to α [85].

According to N. Vidakis et al., PLA/MWCNT nanocomposite materials exhibit thermal stability up to around 270 °C with no measurable weight loss. Between 270 °C and 390 °C, there is a second temperature range where the materials begin to degrade and decompose, with decomposition starting at 273 °C (Ton). In the third range (>390 °C up to 550 °C), all samples of PLA material have completely decomposed [84]. Moreover, Lucia Fama et al. found that the degradation temperature of materials with less than 2% functionalized nanofillers decreased. They determined that the nanocomposites made from a bio-based and sustainable material like PLA and strengthened with a minimal amount of functionalized MWCNT exhibit appropriate characteristics for various sectors like packaging, coatings, medicine, textile, and engineering [86].

D. Wu et al. utilized melt mixing to produce PLA nanocomposites incorporating various functionalized MWCNTs. The existence of carboxylic and purified MWCNTs delays the thermal depolymerization of PLA because of their barrier and thermal conductive properties during the initial degradation stage in all composites [118]. Other studies by F.Z. Yakdoumi et al. developed new functionalized MWCNT for food packaging by modifying them with polydopamine and TiO_2_ nanoparticles. These TiO_2_-PDA-MWCNT were then used as fillers in PLA nanocomposite films to enhance food packaging performance. The nanocomposites showed increased crystallinities compared to PLA, with levels about 10% higher than pure PLA. Among the materials tested, the PLA nanocomposite showed the most impressive mechanical properties, including superior Young modulus and hardness modulus values [119]. According to Lizundia et al. [120], a notable 100% increase in the thermal conductivity of PLA/CNT composites was observed with 3 wt.% CNT. The thermal conductivity decreased when CNT was added at levels up to 0.75 wt.%, attributed to an interfacial resistance causing photon scattering and reducing bulk thermal conductivity. By incorporating more CNT, the composites achieved a denser network of conducting pathways, resulting in an increase in thermal conductivity [121].

In addition, Yarici et al. used different crystallization kinetics models (e.g., Avrami, Ozawa, and Avrami and Ozawa) to evaluate the non-isothermal crystallization behavior of PBS/MWCNT nanocomposites based on surface modification type. They prepared MWCNT nanocomposites (PBS/MWCNT) with different loading levels (0.5 and 1 wt.%) using a twin-screw micro-composition machine. Acid treatment and chemical modifications were applied to the surface of the MWCNTs. Each type of carbonyl acid and each type of alkyl group with different chain lengths were incorporated into the MWCNT surface. Therefore, it is concluded that chemically modified MWCNT nano-encapsulated nanoparticles support the entire non-thermal crystallization process in pure PBS [58].

Wang et al. [122] used simple melt compounding to prepare biodegradable PBS/pristine raw MWCNT composites. The addition of 0.5 wt.% MWCNT to PBS/MWCNT composites resulted in a crystallization temperature of 90 C, approximately 6 °C higher than that of pure PBS. T_c_ values increased marginally with increasing MWCNT content. Thermogravimetric analysis showed that the inclusion of MWCNTs enhanced the thermal stability of PBS compared to pure PBS. In addition, according to the research of Pramoda et al. [123] and Song and Qiu [124], which looked at the crystallization behavior of PBS and MWCNT composite materials formed by melt mixing, MWCNTs may play an important role in heterogeneously nucleating the PBS crystal, thereby increasing the rate of crystallization of the PBS.

In addition, due to the heterogeneous nucleation effect of the nucleation, the addition of SWCNT-APTES enhances the crystallization of PBS in the nanocomposite. Due to the covalent bond after hydrolysis of the SWCNT, the SWCNT is more easily dispersed in the PBS matrix. The crystallization rate of PBS is higher in the PBS/SWCNT-APTES (1% mixture) nanocomposite than in the PBS/SWCNT-APTES (1%) nanocomposite, which may be caused by a reduction in the growth constant after hydrolysis [90].

### Comparative Thermal Properties

Thermal properties of MWCNT-reinforced polyester composites are characterized primarily by their capacity to serve as heterogeneous nucleating agents for crystallization and thermal stability. However, the degree of this enhancement greatly depends on the structural features of the polymer matrix, dispersion of nanotubes, their surface modification and processing techniques rather than on the number of nanotubes.

**Figure 11:**
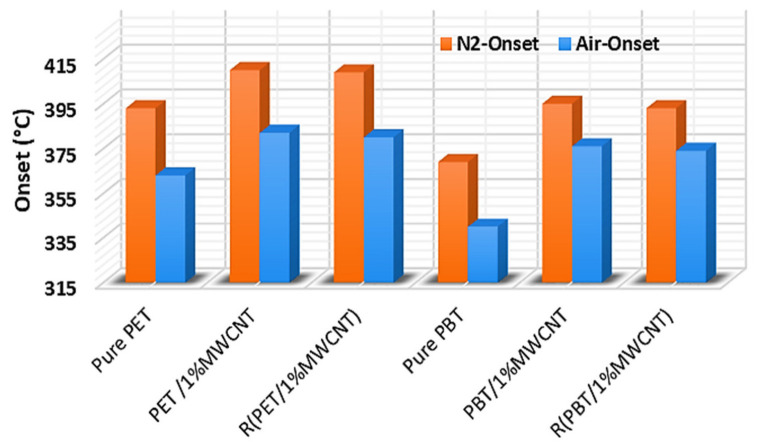
Onset temperatures of PET, PBT, and its nanocomposites [18].

In terms of their thermal stability, PET appears to be the most improved among the four matrices. Its crystallization temperature can increase by up to 25 °C (198–223 °C), cold crystallization is suppressed, and thermal degradation becomes more resistant to about 20–50 °C due to well-dispersed acid-functionalized MWCNT (Table 2) [125]. Such improvements result from enhanced nucleation and restricted polymer chain mobility. On the other hand, too much restriction of chains can become an obstacle for additional processing (annealing and drawing processes). At the same time, the nucleation properties of PBT are like those of PET and include increased crystallization temperature (by 11.2 °C), an increase in crystallinity from 24.5% to almost 39%, and a slight increase in heat deflection temperature (51–55 °C) (Table 2) [97]. A recent study by Khan et al. [18] discovered that the incorporation of MWCNTs increased both the temperatures of the onset and peak of thermo-oxidative degradation of PBT and indicated the higher intrinsic thermal instability of PBT compared to PET without MWCNT incorporation (Figure 11). It is a significant clarification for numerous reviews, which consider all kinds of “engineering polyesters” as equal thermal bases for MWCNT application. Obviously, the inherent thermal stability of the matrix restricts the extent to which the inclusion of a certain filler affects it. Moreover, the lack of independent research into PBT/MWCNT composite materials, which provide the entire range of properties (T_g_, T_c_, T_m_, X_c_, and degradation kinetics), cannot be attributed to any limitations of this review’s methodology but rather constitutes a real literature gap, and future investigations into this issue will help to differentiate between the nucleation effect and the thermal effect of an elastomeric phase.

In the case of PLA, the thermal properties are more complicated due to the interaction of both MWCNTs and plasticizers with the polymer crystallization process. MWCNTs promote β-type crystallization and improve thermal properties (Table 2) [99]. However, quantitative analysis of crystallinity and degradation is usually not provided in the available literature; therefore, comparison with PET and PBT in this respect is difficult. Also, major reductions in glass transition temperature are caused by plasticizers rather than by MWCNTs. The highest but still moderate enhancement is observed in PBS (Table 2) [102]. There is a small increase in crystallization and melting temperatures (about 4–5 °C) irrespective of the processing technique used [102]. However, some limitations must be noted; there are no standardized processing conditions for comparative analysis, insufficient quantitative thermal data in the case of PLA and PBS, and a lack of DSC-TGA data of neat PBT composites.

**Table 2:** Thermal properties of different polyester MWCNT nanocomposites.

Matrix	Filler Type	wt.%	T_g_ (°C)	T_c_ (°C)	T_m_ (°C)	Crystallinity Effect	T_5%_ or Tonset (°C)	T_max_ (°C)	Major Findings	Ref.
PET	MWCNT	1	Not reported	Not reported	Not reported	Not reported	363 -> 385 (+22 C onset, N2)	445 (DTG peak)	MWCNT addition raised both the onset and DTG-peak degradation temperatures of PET.	[18]
PET-	MWCNT-COOH	1.5, 2, 3, 6	81 -> 80 (1.5–2 wt.%)	198 -> 218 (1.5–2 wt.%)	252 -> 248–249 (down 3–4 C, all loadings)	10% -> 24.7, 23.6, 36.4, 36.3% (+26.4 pts at 3 wt.%)	Not reported	Not reported	At 3 wt.% and above no T_g_ was detectable, indicating hindered chain dynamics.	[125]
PET	MWCNT-COOH	0.1, 0.5, 1	Not reported	Not reported	Not reported	Not reported	396 -> 401 (0.1 wt.%) -> 405.7 (0.5 wt.%) -> 418 (1 wt.%) (+22, N2)	440 -> 458.5 -> 465.7 -> 488 (+48 C at 1 wt.%)	Residual char yield at 600 C rose from 19.5% (neat) to 35% (1 wt.%), attributed to MWCNT acting as a physical/crosslinking barrier to degradation.	[75]
PBT	MWCNT	1	Not reported	Not reported	Not reported	Not reported	->376 (onset, air)	415 (DTG peak, air)	MWCNT raised the thermo-oxidative onset and peak degradation temperatures of PBT	[18]
PBT	MWCNT	1–5 phr	59–61	(blend) -> 203.6, 204.6, 206.1, 206.3, 207.3	(blend) -> single peak ~221.6–222.5	(blend) -> 38.4, 38.3, 33.3, 28.6, 38.9%	Not reported	Not reported	MWCNT addition eliminated the double melting peak seen in neat PBT (more uniform crystallization) and raised T_c_ by up to 11.2 °C	[97]
PLA	MWCNT	0.5, 1, 3, 5	Not reported	Not reported	Not reported	Increased with CNT loading	Higher onset vs. neat PLA	Highest stability at 5 wt.% CNT	MWCNTs progressively raised and improved thermal stability at every loading tested, with 5 wt.% CNT	[99]
PBS	MWCNT	0.5–3.0	Not reported	Increased by 4 (3 wt.%)	Increased by 5 (3 wt.%)	Not reported	Not reported	Not reported	MWCNT nucleated PBS crystallization, raising both T_c_ and T_m_ modestly at 3 wt.%, versus neat PBS.	[102]

“->” indicates the effect of the MWCNT modification on the value of the given property.

## 6. Electrical Properties

W. Aloui et al. [126] observed that higher concentrations of carbon nanotubes lead to lower sheet resistance values and that thicker films demonstrate improved electrical characteristics because of the greater number of electron pathways available for travel. The key factors that can be attributed to the outstanding intrinsic conductivity and high crystallinity of MWCNTs are the percolation threshold, which allows carriers to move between tubes in the film, and the concentration and distribution of MWCNTs on the PET substrate. Thin films that are both highly conductive and transparent were fabricated using extremely purified MWCNTs [127,128]. According to Avilés et al., [59] electrical percolation occurred at a rate of about 1 wt.% for composites treated by direct extrusion and melt compounding followed by extrusion, and electrical conductivity after percolation was higher for composites created by melt compounding. At 9 wt.%, melt compounding composites have a mean electrical conductivity of 1.54 Sm^−1^, whereas direct extrusion composites have a conductivity of 0.45 Sm^−1^ (Table 3) [59].

Moreover, Choi et al. [129] studied the electrical conductivity of PBT composites at different levels of MWCNT content. MWCNTs were modified via physisorption and then blended with PBT to create composites through melt extrusion. The electrical conductivity underwent a sudden increase when the PBT composites were incorporated with 1.0 wt.% of pure MWCNT and 0.75 wt.% of PBT-PBOH-MWCNT. Chemical treatment of MWCNTs leads to the formation of defects that negatively impact the electron transport within the MWCNT structure. This implies that the PBT/MWCNT composite with the highest reinforcement, fatigue life, and electrical conductivity was created using PBOH-MWCNT through reactive extrusion [57]. Additionally, N. Vidakis et al. examined the electrical conductivity of PLA/MWCNT nanocomposites with 1.0, 2.5, and 5.0 wt.% MWCNT in the nanocomposites. The electrical conductivity is measured in two different directions: “through layer” and “cross layer”. Increasing the loading of CNT conductive filler was discovered to result in higher electrical conductivity in both directions [84]. According to Kim et al. [130], PLLA and MWCNT electrical resistivity decreased steadily as the MWCNT concentration increased compared to its PLLA counterpart containing MWCNTs. This was due to the inhibition of direct PLLA-g-MWCNT interactions.

The PLLA/MWCNT composites were characterized by Lizundia et al. [131] using the solvent casting method. MWCNTs were randomly distributed within the polymer matrix, with a physically continuous pathway formed at concentrations of MWCNT 0.25 and 0.5 wt.%. The percolation threshold for MWCNT ranged from 0.21 to 0.33 wt.%, and the conductivity increased by six orders of magnitude. Moreover, Li et al. [132] fabricated PLA/C-MWCNT through in situ polymerization. The addition of C-MWCNT significantly improves the electrical conductivity in PLA. Another study fabricated PLA/MWCNT composites with varying MWCNT contents (0.5 to 2.0 wt.%) through a melt mixing process; the electrical percolation threshold was obtained below 0.5 wt.% of MWCNT content, which is associated with the formation of a conductive network structure in the PLA matrix [133].

According to Sinha Ray et al., the electrical conductivity of pure PBS increased by 10^6^ times when the nanocomposite was melt-blended in a batch mixer, and the MWCNT loading was 3 wt.%. In plane, the conductivity increased from 5.8 × 10^−9^ S/cm for pure PBS up to 4.4 × 10^−3^ S/cm for nanocomposite [89].

Y.F. Shih et al. found that surface resistivity decreased as the nanotube content increased, but not as much as in PBS/u-CNT composites. This suggests that CNT is distributed more evenly throughout the polymer matrix than u-CNT. MWCNTs in polymer matrices can bond with each other to form an interconnected conductive channel, allowing a very large percentage of electrons to flow through the sample when an electric field is applied [91].

Thus, petroleum-based PET/MWCNT nanocomposites normally demonstrate better electrical behavior due to strong interface interactions between aromatic PET chains and the graphitic surface structures of nanotubes [134,135]. The above literature shows that interconnected MWCNT network development in PET can reduce its electrical resistivity by several orders of magnitude. Consequently, such materials could be used for antistatic, sensor, and EMI shielding applications. In turn, low percolation thresholds and high electrical conductivity have been observed for PBT/MWCNT nanocomposites, which is explained by rapid crystallization facilitating MWCNT localization and forming conductive pathways [136]. In contrast, bio-based PLA and PBS polymers are insulators but become electrically conductive with the use of MWCNTs. The conductivity required for electrostatic discharge protection and flexible electronics can usually be obtained in relatively small amounts of MWCNTs for PLA [137]. Significant conductivity enhancement in PBS/MWCNT composites is observed as well, allowing for new opportunities in EMI shielding and green electronics [138]. It can be noted that nanocomposites based on PET and PBT show better electrical conductivity and lower percolation thresholds compared to PLA- and PBS-based polyesters due to higher nanotube dispersion and stronger interaction between matrix and fillers. However, bio-based PLA/MWCNT and PBS/MWCNT nanocomposites offer environmentally friendly solutions for conductive polymers while maintaining satisfactory electrical properties.

### Comparative Electrical Properties

Electrical conductivity of MWCNT-filled polyester composites is largely driven by the concept of electrical percolation, which is controlled to a greater extent by nanotube dispersion, interfacial properties, and processing approach rather than the polymer matrix. The threshold value of percolation, reported across PET, PBT, PLA, and PBS, ranges from 0.25 to 14 wt.% of MWCNTs, indicating that the processing technique is the main driving force of the formation of a conducting network. Among all four polyesters, PET shows the highest stability and reproducibility of electrical characteristics and has the lowest percolation thresholds in the range of 0.33–1 wt.% of MWCNT depending on the processing procedure. Coagulation processing and improved melt mixing result in an even distribution of MWCNT and conductivity, while over-functionalization with acids leads to degradation of the conjugation system of MWCNT, resulting in decreased conductivity despite the improved interface adhesion [59,92,139]. In turn, a significant increase in conductivity is achieved for PBT only in the case of interfacial engineering. Reactive ionic liquid compatibilizers used in conjunction with carboxylated MWCNT resulted in approximately a 10-fold increase in conductivity [97,140].

**Figure 12:**
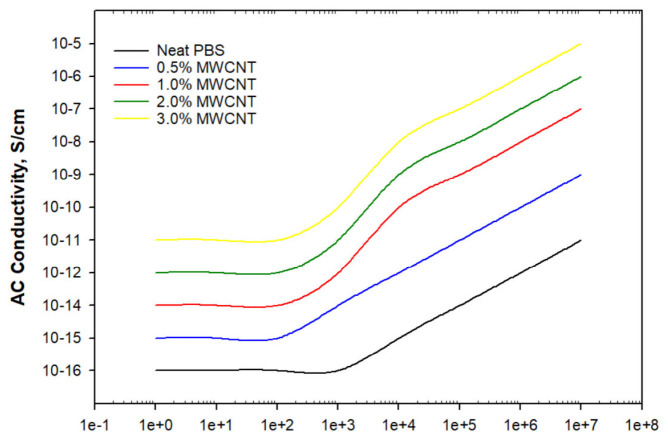
The AC conductivity of electrospun MWCNT/PBS nanocomposite material [102].

In contrast to PET and PBT, PLA has the highest variation in its electrical characteristics, with the percolation threshold in the range of 1–14 wt.% of MWCNT depending on dispersion characteristics. Biodegradable lignin dispersant provides the greatest improvement of conductivity (by 6 orders of magnitude) when the content of MWCNTs is constant, while conductivity might be decreased above the optimal content because of increasing crystallinity (Table 3) [141,142,143]. Despite the much lower number of studies on PBS, PBS has one of the lowest percolation thresholds (<1 wt.%) (Table 3) using electrospinning, which results in highly conductive networks and EMI shielding (Figure 12) [102].

Thus, the important finding is that processing procedures and interfacial design have a greater impact on electrical conductivity than the choice of polymer or amount of filler. However, there is still a need to investigate some issues, like the limited number of studies devoted to the electrical characteristics of PBT, the absence of standard processing conditions that allow for comparison of conductive properties of different polyester matrices, and the lack of knowledge on the combined effect of nanotube functionalization and crystallinity.

**Table 3:** Electrical properties of different polyester MWCNT nanocomposites.

Matrix	Filler Type	wt.%	Electrical Conductivity	Major Findings	Ref.
PET	MWCNT, Treated (T-MWCNT)	0–2	Percolation reached at 0.33 wt.% (σ ≈ 2 × 10^−3^ S/cm, 0.2 S/m); T-MWCNT (surface-treated) stayed non-conductive up to 2 wt.%	Pristine MWCNTs greatly outperform acid/oxidatively treated MWCNTs for conductivity at equal or higher loading.	[92]
PET	MWCNT	9	MCE conductivity (1.54 × 10^−2^ S/cm) ≈ 3.4× higher than DE (0.45 × 10^−2^ S/cm)	Percolation threshold ≈ 1 wt.% for DE and MCE; MCE gave the most homogeneous MWCNT dispersion and highest conductivity.	[59]
PBT	MWCNT (in PBT/POE-g-GMA blend)	4.5	≈4 orders of magnitude (≈4.5 × 10^3^-fold) vs. neat PBT (σ = 2.37 × 10^−11^ S/cm); composite reaches σ = 1.06 × 10^−7^ S/cm	Conductivity rise remains below full percolation.	[97]
PBT	Carboxylated MWCNT + epoxy-ionic-liquid (EPIL)	0.7 (+2.0 wt.% EPIL)	≈10 orders of magnitude vs. neat PBT (σ = 2.24 × 10^−16^ S/cm); composite reaches σ = 1 × 10^−6^ S/cm	Very low percolation threshold (0.7 wt.% c-MWCNT) compared with unmodified c-MWCNT/PBT systems.	[140]
PLA	MWCNT-PLLA	14	Conductivity reaches ≤0.1 S/cm at highest loading	DC conductivity rises with loading following percolation behavior.	[142]
PLA	CNT PLLA	1	Conductivity peaks at 1 wt.% CNT (σ ≈ 4.4 × 10^−2^ S/cm), then decreases at 2 and 5 wt.%	Conductivity declines above 1 wt.% attributed to CNT-induced PLA crystallization restricting chain mobility/network quality	[143]
PBS	MWCNT	0.94, 2	≈9 orders of magnitude vs. neat PBS (σ = 7.8 × 10^−14^ S/cm); composite reaches σ ≈ 1.9 × 10^−1^ S/cm at 2 wt.%	Electrospinning promotes MWCNT alignment and uniform distribution, enabling an effective percolation network at low loading (0.94 wt.%).	[102]

## 7. Recycling

The environmental impacts of polyester are significant because most polyesters are non-biodegradable. However, the negative impact of polyester on the environment in recent years has been significantly decreased due to technological advancement within related industries. Due to increasing environmental concerns and expanding legislation on recycling and sustainability at the international level, there has been a tremendous growing trend towards sustainable and environmentally friendly products [144].

The second most recycled thermoplastic after polypropylene is PET. The addition of MWCNTs to recycled PET (rPET) has recently attracted substantial interest as a promising way to develop advanced materials from otherwise wasted PET [145]. Mechanical recycling typically leads to scission and a loss of molecular weight and mechanical properties. Therefore, reinforcing recycled polymers with MWCNTs might compensate for these drawbacks. Among the earliest works investigating PET/CNT composites, one should mention research conducted by Molnár et al. [146]. In this paper, the authors explored MWCNT-reinforced composites produced from mechanically recycled PET bottle waste. It was found that proper cleaning and processing of PET scraps considerably affected the rheology and mechanical properties of resulting composites. The authors also reported that introduction of coupling agents facilitated better dispersion of nanotubes and more efficient stress transfer between the rPET matrix and MWCNTs. A significant step in the investigation of PET/MWCNT composites was made by Chowreddy et al. [147]. The researchers managed to produce recycled PET/MWCNT nanocomposites using masterbatch dilution technology with twin-screw extrusion. They observed that at just 0.25 wt.% MWCNT content, there was a significant increase in melt viscosity, storage modulus, and loss modulus. This was attributed to the formation of an MWCNT network in the polymer matrix. Nanotube presence also increased crystallization temperature and extent due to heterogeneous nucleation. Tensile strength and tensile modulus increased compared with neat rPET, whereas thermal stability and glass transition temperature showed only a marginal rise. Therefore, it was shown that MWCNT could turn rPET into a high-performance engineering plastic (Table 4).

Furthermore, Fang et al. [148] investigated blends of recycled PET and thermoplastic polyurethane, reinforced with MWCNT. The researchers found that the nanotubes increased the mechanical performance, thermal stability, and crystallization properties. The researchers concluded that the nanotube addition was capable not only of overcoming PET recycling drawbacks but also of increasing interfacial adhesion between polymer phases in the system. Apart from engineering applications, PET-based MWCNT nanocomposites have also been tested for environmental use. Mallakpour et al. [149] created nanocomposites using a PET bottle scrap as the polymer matrix. As a result, nanocomposites featured higher thermal stability and electrical conductivity while being capable of absorbing Cd(II) ions from solution. Thus, it became clear that recycled PET scraps might serve as a base for the creation of useful materials.

It should be noted that despite the abundance of works dedicated to PET matrix recycling, there are rather few papers devoted to repeated recycling of PET/MWCNT systems. Based on the experience of other CNT-reinforced thermoplastics, it may be concluded that multiple recycling would help maintain mechanical and rheological properties due to nanotubes compensating polymer degradation. However, during recycling, nanotubes can suffer from shortening, modification of network structure, and even changes in electrical conductivity. Consequently, studying the recyclability of PET/MWCNT will remain a relevant question in the future. Also, it should be mentioned that PET/MWCNT systems can face the problem of thermal degradation during recycling [150].

Unlike PET, the recycling of PBT has not garnered as much attention due to lower production rates and its application in durable items rather than disposable packaging materials. Despite this, the incorporation of MWCNTs into PBT raises issues regarding the recyclability and long-term environmental impact of such materials. Among the major studies concerning MWCNT-loaded PBT, one can highlight the study carried out by Socher et al. [151], which explored the effect of matrix viscosity on the dispersion of MWCNTs and their electrical characteristics in various engineering thermoplastics, such as PBT, polyamide 12, polycarbonate, PEEK, and LDPE. It was revealed that the viscosity of the matrix had a major impact both on the percolation threshold of the material and on the agglomerate state of MWCNTs. In addition, the matrix viscosity and conditions of melting had a substantial influence on both the stability of the network structure and its dispersion quality.

It should also be mentioned that the thermal stability of PBT/MWCNT compounds can play a significant role in the recycling process because CNTs increase the temperature at which the polymer starts to degrade and serve as a barrier slowing down the diffusion of volatile products. According to Kim et al. [76], conductive CNT networks are known to improve the thermo-oxidative stability of engineering thermoplastics. Consequently, they may help preserve the performance of materials when recycled. Thermal stability is especially important for PBT because of high melting temperatures that usually reach values ranging from 240 to 270 °C.

Unfortunately, very few studies devoted to the investigation of recycled PBT/MWCNT nanocomposites exist. Thus far, the recycling of PBT/MWCNT compounds has remained an underdeveloped field of research. The existing information is largely indirect and comes from processing, rheological, conductivity, and thermal stability studies [18]. Clearly, more research is needed to determine what happens to mechanical and electrical properties, how much nanotubes may degrade during multiple processing cycles, and whether it affects environmental issues (Table 5).

PLA is a bio-based and industrially compostable polyester. Many works have been dedicated to improving the thermal stability, brittleness, and low electrical conductivity of PLA through the addition of MWCNTs. However, the presence of MWCNTs influences both mechanical recyclability and degradation properties. The recent study by Kaczor et al. [152] is among the very few investigations that have directly studied the multiple reprocessing behavior of highly filled PLA/MWCNT composites (up to 25 wt.% CNT) through the simulation of recycling via multiple extrusion using both single- and twin-screw extruders. The results of this study have shown that repetitive extrusions caused a gradual reduction in processing torque and efficiency, which was mainly due to a reduction in the viscosity of PLA resulting from chain scissions. DSC confirmed that cold crystallization temperature decreased whereas cold crystallization enthalpy increased significantly for all reprocessed cycles, and the melt flow rate increased consistently, thus revealing the matrix degradation effect. Crucially, no changes in the MWCNT structure could be seen in both XRD and Raman analyses following the three extrusion cycles, indicating that the nanotubes were relatively stable during reprocessing while the PLA matrix degraded.

In another recent research work by Silva et al. [153], the authors fabricated PLA/PBAT-g-GMA/MWCNT nanocomposites intended for electrostatic dissipation applications. The thermoplastic nature of PLA and PBAT-g-GMA in theory allows reuse via standard mechanical recycling procedures (extrusion or injection molding); an investigation into the stability of the conductive network and mechanical behavior of these nanocomposites during multiple reprocessing cycles is yet to be done (Table 4 and Table 5). Such studies show the overall trend in this area, namely most PLA/CNT studies determine the basic thermal, rheological, and electrical properties of these materials [154]. Although there is a lack of literature on the mechanical recyclability of PLA/MWCNT nanocomposites, data obtained when studying the reprocessing of polymer nanocomposites shows that nanofillers partly mitigate the effect of chain scission during repeated extrusion of materials [155].

The recycling literature on PBS/MWCNT nanocomposites is much less abundant than the literature on their PLA counterparts. Most of the research in this area [91] is by Shih et al., who first introduced melt-blended PBS/MWCNT nanocomposites with surface-modified nanotubes providing for about a six-order-of-magnitude increase in electrical conductivity compared to neat PBS. The study was mostly devoted to virgin material preparation, filler dispersion, and property enhancement (mechanical, thermal, electrical). Similarly, more recent research on PBS/MWCNT nanocomposites [103] focuses on masterbatch-based fabrication methods allowing for the control of filler dispersion and improvement of the mechanical, tribological, and thermal properties of the materials [156].

Additionally, the reprocessing behavior of the PBS matrix without any filler was investigated in Gigante et al. [157], where a production scrap of PBS was subjected to five consecutive recycling cycles through semi-industrial twin-screw extrusion (Table 4). The results of this experiment showed that the melt mass-flow rate progressively increased in each cycle, while DSC and TGA analyses revealed only minor changes in glass transition, crystallization, and the degradation temperatures of the material. This suggests that PBS is relatively sustainable during mechanical reprocessing through multiple extrusions under conventional extrusion conditions, much lower than its degradation onset temperature. Still, this study did not use MWCNT or any other nanofillers, leaving the question open as to the behavior of the CNT percolated network and the possibility of its sustainability under the same conditions. There is very little literature describing the repeated extrusion, injection molding, or chemical recycling of PBS/MWCNT nanocomposites. At this point, there is a lack of information about the recycling processes since all the data comes from thermal stability or crystallization kinetics experiments (Table 3).

In the recycling of polyesters, thermal degradation involves the molecular chains of the polymer bonding with water molecules to form an ester link and then starting to dissociate it. Heat is primarily required, and residues, ash, and incombustible gases are the end products. When the entire procedure is finished, a polyester condensation reaction with the hydroxyl group in the waste material reconvening with the carbonyl group of the ester link is sustainable, and the formation of water is observed [158,159].

Similarly, microbes have the capability to assay and knock down the molecular weight of the polyester waste in the process of biodegradation. This has been shown to be possible with laboratory-scale worm, fungi, and bacterial actions, but the rate and extent of the biodegradation are determined by various waste and environmental conditions. The waste remains in the original form but with reduced molecular weight, and the potential to become biomass is enhanced [160,161].

**Table 4:** Recycling properties of different polyester MWCNT nanocomposites.

Matrix	Filler Type	Major Findings	Research Gaps	Ref.
PET (rPET)	MWCNT	Scrap cleaning/processing governs rheological and mechanical performance; coupling agents enhance MWCNT dispersion and interfacial stress transfer.	Limited data on multiple-recycling behavior; unclear effects on nanotube integrity, network structure, conductivity, and thermal degradation risk over repeated processing.	[146]
PET (rPET)	MWCNT	Increases melt viscosity, storage/loss moduli, and crystallization; improves tensile strength/modulus with only marginal gains in thermal stability and T_g_.		[147]
PET (bottle scrap)	MWCNT	Enhances thermal stability and electrical conductivity; imparts Cd(II) adsorption capacity for environmental remediation use.		[149]
PBT	MWCNT	Conductive CNT networks improve thermo-oxidative stability, potentially preserving properties through recycling; particularly relevant given PBT’s high melt temperature (240–270 °C).	Recycling of PBT/MWCNT remains largely unexplored; existing evidence is indirect, drawn from processing and property studies rather than actual recycling trials.	[18,76]
PLA	MWCNT (up to 25 wt.%)	Repeated extrusion reduces processing torque and viscosity via matrix chain scission; cold crystallization temperature decreases while enthalpy and melt flow rate increase, confirming matrix degradation.	Mechanical recyclability underexplored; conductive network/mechanical stability under repeated reprocessing not established. Available nanocomposite evidence suggests fillers may mitigate chain scission.	[152]
PLA/PBAT-g-GMA	MWCNT	Nanocomposite developed for electrostatic dissipation; theoretically recyclable via standard mechanical methods.		[153]
PBS	-	Five recycling cycles show progressively rising melt flow rate but minimal changes in T_g_, crystallization, and degradation temperature, indicating good reprocessing stability.	No recycling data for PBS/MWCNT specifically; unknown whether MWCNT percolated network remains stable under repeated reprocessing; available evidence limited to thermal/crystallization studies.	[157]

**Table 5:** Comparative summary of petroleum-based and bio-based polyester MWCNT nanocomposites.

Property	Petroleum-Based (PET, PBT)	Bio-Based (PLA, PBS)
Processbility	Established melt-processing routes (extrusion, injection molding) with mature industrial infrastructure [162].	Compatible with melt blending, solution casting, and FDM filament extrusion, though dispersion remains more sensitive to processing route [163].
Mechanical	MWCNT content increases can enhance mechanical properties by up to 40%, while processing parameters alone shift performance by ~30% at fixed filler content [164].	Plasticized PLA/MWCNT systems show ~20% increases in tensile strength, though impact resistance can fall by as much as 80% without plasticizer [100].
Thermal	High thermal and thermo-oxidative stability; delayed degradation onset and higher char yield reported for CNT-reinforced systems generally [153].	MWCNT addition reduces PLA glass transition and melting temperatures by up to 9.4 °C and 23.3 °C, respectively, while introducing cold crystallization [165].
Electrical	Conductive networks form readily in PBT-based systems compatibilized with elastomeric phases, supporting EMI shielding and electrostatic dissipation applications [97].	PLA/MWCNT composites reach percolation-driven conductivity suitable for 3D-printed conductive parts, with resistance decreasing as MWCNT content increases [165].
Recyclability	Mechanically recyclable via established PET/PBT streams, though MWCNT–nanocomposite-specific recycling behavior is less studied [18].	Biodegradable under appropriate conditions (e.g., PLA requires industrial composting ≥ 58 °C); biodegradation rate in PBS/MWCNT systems increases with filler content above a threshold [19].

## 8. Conclusions

The structure of polyester polymer molecules determines the properties of the material. The short ethylene glycol in PET makes it more rigid, gives it a high glass transition temperature, and makes it dimensionally stable and thus more suitable for strength and barrier applications. On the other hand, the longer butylene glycol in PBT increases chain flexibility and the crystallization rate and is thus suitable for engineering parts that require toughness and quick processability. With aliphatic polyesters, the methyl groups on PLA make it more rigid and strong and thus less flexible; thus, it has a higher glass transition temperature. PBS has a flexible backbone and is hence more ductile and tough and crystalizes faster.

Petroleum-based polyesters have been mainly manufactured by melt compounding and masterbatches due to the level of maturity in these technologies for industrial purposes. However, bio-based polyesters will be greatly assisted by solution approaches due to their ability to improve the dispersion of nanotubes and improve interfacial compatibility. Even though in situ processes are known to yield the best matrix–filler interaction and distribution, their high cost of production makes them not suitable for large-scale commercialization. It is anticipated that future research will explore hybrid processes that merge both melt and solution approaches.

Petroleum-based polyesters display better mechanical properties than PLA and PBS. Due to its aromatic molecular structure and high crystallinity, PET possesses excellent stress transfer ability with MWCNTs, leading to high levels of tensile and flexural strength, especially in functionalized nanotube-filled PET. The fast crystallization and intrinsic stiffness of PBT confer outstanding flexural properties. In contrast, the PLA/MWCNT composites are moderately improved in terms of strength and Young’s modulus while being toughened due to decreased brittleness with the help of crystallization promotion. PBS composites feature weaker strength because of flexible aliphatic chains, yet they have better ductility and impact resistance.

In terms of thermal stability, petroleum-based nanocomposites feature high stability due to their aromatic nature and strong interfaces between the matrix and nanotubes. For bio-based polyesters, MWCNTs provide higher crystallinity and thermal stability, which limit chain mobility and promote crystallization. Even though PET and PBT nanocomposites are thermally resistant, PLA and PBS utilize MWCNTs to increase crystallization rates and dynamic behavior. Furthermore, the electrical properties of PET and PBT nanocomposites have an advantage over those of bio-based nanocomposites because of higher interfacial interactions and dispersion of the nanotubes, resulting in a lower electrical resistivity and percolation threshold. Such properties can be used in the manufacturing of antistatic materials, sensors, and electromagnetic interference (EMI) shields. PLA and PBS nanocomposites are intrinsically insulating but become electrically conductive with the addition of MWCNT. Thus, petroleum-based nanocomposites are the perfect materials for the manufacture of high-performance engineering products due to their mechanical, thermal, and electrical properties. Bio-based nanocomposites can serve as sustainable and environmentally friendly materials with enhanced properties.

The recyclability of thermoplastic polyester MWCNT nanocomposite has remained unexplored. Although PET polyester recycling has received extensive attention, the process of recycling PET/MWCNT nanocomposites poses many problems, such as a reduction in the length of nanotubes and a decrease in electrical conductivity. On the other hand, there is no extensive study on the recyclability of PBT/MWCNT nanocomposites. Some stability of nanotubes has been noted in the case of PLA/MWCNT, whereas recycling of PBS/MWCNT was not studied integrally.

## Data Availability

No new data were created or analyzed in this study. Data sharing is not applicable to this article.

## Abbreviations

The following abbreviations are used in this manuscript:

PET	Polyethylene terephthalate
PBT	Polybutylene terephthalate
PEN	Polyethylene naphthalate
PTT	Polytrimethylene terephthalate
PLA	Polylactic acid
PBS	Polybutylene succinate
PHAs	Polyhydroxyalkanoates
PEF	Polyethylene Furanoate
MWCNT	Multi-walled carbon nanotubes
PLLA	Poly-L-lactic acid
CNT	Carbon nanotubes
EMI	Electromagnetic interference
rPET	Recycled PET
DSC	Differential scanning calorimetry
XRD	X-ray diffraction
PBAT	Polybutylene adipate terephthalate
GMA	Glycidyl methacrylate
TGA	Thermogravimetric analysis
